# Primary care micro-teams: an international systematic review of patient and healthcare professional perspectives

**DOI:** 10.3399/BJGP.2022.0545

**Published:** 2023-08-08

**Authors:** Charles Coombs, Tanya Cohen, Claire Duddy, Kamal R Mahtani, Emily Owen, Nia Roberts, Aman Saini, Alexander Staddon Foster, Sophie Park

**Affiliations:** University College London, London.; University College London, London.; University of Oxford, Oxford.; University of Oxford, Oxford.; University College London, London.; University of Oxford, Oxford.; University College London, London.; University College London, London.; University College London, London.

**Keywords:** continuity of care, family practice, general practice, humans, multidisciplinary, team

## Abstract

**Background:**

International trends have shifted to creating large general practices. There is an assumption that interdisciplinary teams will increase patient accessibility and provide more cost-effective, efficient services. Micro-teams have been proposed to mitigate for some potential challenges of practice expansion, including continuity of care.

**Aim:**

To review available literature and examine how micro-teams are described, and identify opportunities and limitations for patients and practice staff.

**Design and setting:**

This was an international systematic review of studies published in English.

**Method:**

Databases (MEDLINE, EMBASE, CINAHL, Cochrane Library, and Scopus) and grey literature were searched. Studies were included if they provided evidence about implementation of primary care micro-teams. Framework analysis was used to synthesise identified literature. The research team included a public contributor co-applicant. The authors conducted stakeholder discussions with those with and without experience of micro-team implementation.

**Results:**

Of the 462 studies identified, 24 documents met the inclusion criteria. Most included empirical data from healthcare professionals, describing micro-team implementation. Results included characteristics of the literature; micro-team description; range of ways micro-teams have been implemented; reported outcomes; and experiences of patients and staff.

**Conclusion:**

The organisation of primary care has potential impact on the nature and quality of patient care, safety, and outcomes. This review contributes to current debate about care delivery and how this can impact on the experiences and outcomes of patients and staff. This analysis identifies several key opportunities and challenges for future research, policy, and practice.

## INTRODUCTION

While populations increase, the number of general practices continues to decline.^1^ This has instigated a trend towards increased registered patient lists in each general practice.^2^^,^^3^ The belief is that larger interdisciplinary teams can improve access and provide more cost-effective services to patients.^4^^–^^9^ With this expansion of registered patient numbers in each general practice, there is a potential threat that the continuity of care (that is, care that is consistent, patient centred, and holistic)^10^^,^^11^ traditionally experienced in primary care may be lost.^3^^,^^12^^,^^13^ The benefits of larger practice sizes are ambiguous given the limited evidence that clinical outcomes or patient experience can improve.^13^^–^^15^

Continuity of care has been well documented to reduce both mortality and morbidity in addition to a reduction in secondary care referrals.^16^^–^^19^ Lack of continuity may lead to worsened clinical and economic outcomes. Continuity from a specific clinician should improve knowledge of a patient’s personal circumstances and psychosocial history. Despite the perceived benefits, continuity of care has experienced a decline.^20^^,^^21^

The introduction of micro-teams has been proposed to mitigate some of the challenges resulting from practice expansion, to maintain an improved level of continuity in patient care. ‘Micro-team’ is a term introduced in the UK to encourage the organisation of mini-multidisciplinary teams that may serve a particular patient group within the practice (that is, micro-teams within the wider multidisciplinary practice team).^22^^,^^23^ In conjunction with a named GP, patients can develop long-term relationships with several members of a multidisciplinary team. Alongside the established roles in general practice such as nursing and pharmacy, the team can include emerging roles, including physician associates, occupational therapists, physiotherapists, dieticians, health coaches, and paramedics.^22^^–^^27^ The novelty of micro-teams has meant there is flexibility regarding which roles are incorporated into the team. An illustrated depiction of micro-teams is included in Supplementary Figure S1.

## METHOD

This systematic review aims to review the available literature to examine how micro-teams are described and the opportunities that primary care micro-teams can provide for practice staff and patients, and limitations to their introduction and implementation.

**Figure 1.**
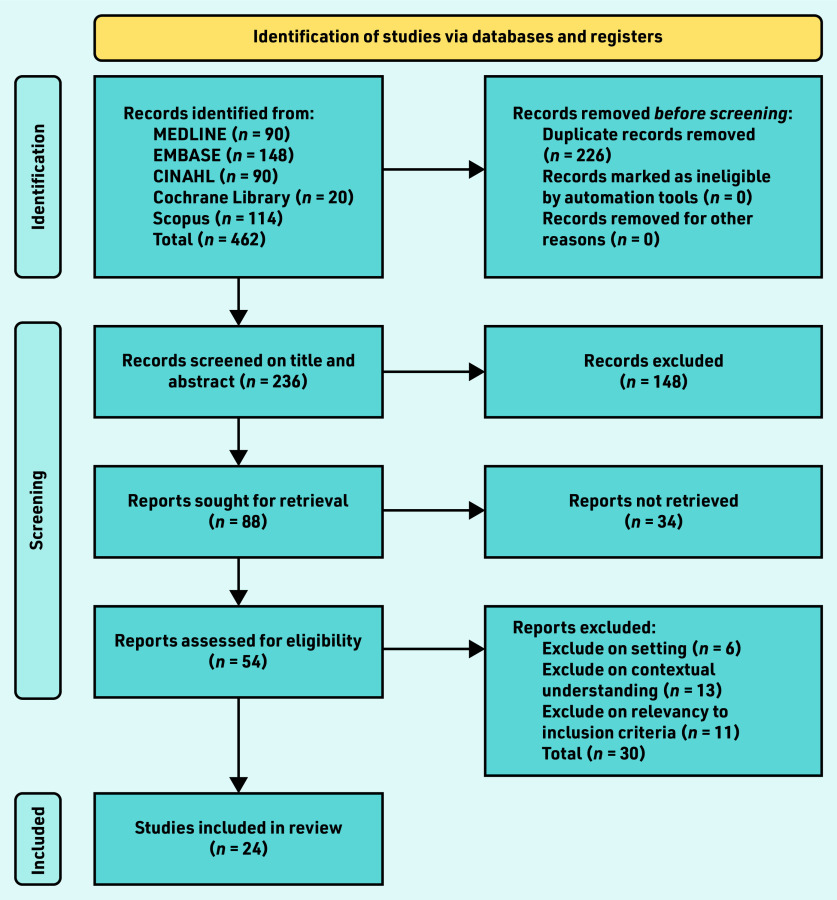
*PRISMA flow chart. Reports were ‘not retrieved’ if the full text was not obtainable. Authors were contacted for any missing or incomplete information required to determine inclusion. If there was no response from any viable methods of communication within 4 weeks, the literature was excluded as ‘reports not retrieved’.*

The full methodological steps for this review are published in the review protocol.^28^ The review was conducted between October 2020 and May 2022, with searches run in November 2020. A PRISMA diagram outlining the selection process can be found in Figure 1 and a list of search terms and database results in Supplementary Table S1.

**Table:** How this fits in

The number of GP practices in the UK has overall reduced, while individual practice size lists have increased. This systematic review used a framework analysis to synthesise the current literature available around micro-teams as a potential intervention to mitigate compromised care in larger practices. This review highlighted micro-teams as a structure of general practice to promote accessible healthcare delivery and moderate losses to continuity. Further research into whether continuity can be offered by a team instead of an individual is warranted in the implementation of micro-teams.

A framework analysis approach was used to extract and synthesise data. Deductive analysis explicitly addressed predetermined research questions. Inductive analysis then enabled the authors to respond to the emergent and sometimes unexpected themes identified within the data.^29^ The protocol of this review was registered on PROSPERO (CRD42021225367).

A patient and public involvement collaborator is one of the authors (the second author). This author has been involved from the inception, in the development and review of the protocol, and has been closely involved in the emergent finding stages and iterative analysis throughout the review. Stakeholders were involved in the research as context experts and included a range of GPs, physician associates, primary care network committee members, and practice managers. They provided input to help focus the review, interpret data, and critically discuss emergent findings.

## RESULTS

In total, 24 documents were included in this review (Table 1).^26^^,^^30^^–^^52^ Documents largely referred to US-based healthcare systems (*n* = 18). Most papers were empirical (*n* = 21), including a range of research participants. The remainder were discursive (*n* = 4) and contributed to the theoretical debate about the composition and organisation of micro-teams.

**Table 1.** Characteristics of the literature

**First author**	**Year**	**Country**	**Subject of research, S or P**	**Methods**
**Abrahamson^46^**	2020	UK	S	Mixed methods (primary and secondary qualitative data)
**AuYoung^30^**	2015	US	S + P	Mixed methods (survey + interview)
**Bodenheimer^31^**	2007	US	N/A	Discursive
**Bodenheimer^32^**	2016	US	N/A	Discursive
**Caplan^50^**	2014	US	S	Qualitative
**Chen^33^**	2010	US	S + P	Quantitative
**Contandriopoulos^52^**	2018	Canada	S	Mixed methods (qualitative and quantitative)
**Forman^34^**	2014	US	S	Qualitative
**Funk^35^**	2017	US	S + P	Qualitative
**Gale^36^**	2015	US	S	Quantitative
**Giannitrapani^37^**	2019	US	S	Qualitative
**Harrod^38^**	2016	US	S	Qualitative
**Helfrich^39^**	2014	US	S	Quantitative
**Hofer^48^**	2019	Australia	N/A	Discursive
**Janamian^49^**	2014	Australia	S	Qualitative
**Janamian^51^**	2014	Australia	S	A systematic review (qualitative)
**Jay^40^**	2015	US	S	Qualitative
**Ladebue^41^**	2016	US	S	Qualitative
**Laing^42^**	2008	US	S + P	Mixed methods (quantitative survey and qualitative interviews)
**Ngo^43^**	2010	US	S	Qualitative (vignettes)
**Pandhi^47^**	2018	US	S	Mixed methods (quantitative survey and qualitative interviews)
**Risi^26^**	2015	UK	S + P	Mixed methods (qualitative + article review)
**Rodriguez^44^**	2014	US		
	S	Mixed methods (quantitative survey and qualitative interviews)		
**Rodriguez^45^**	2015	US	S	Mixed methods (quantitative survey and qualitative interviews)

*N/A = not applicable. P = patient. S = staff.*

### Question 1. How are micro-teams described?

The ways in which micro-teams were described and the context for their implementation is summarised in Supplementary Table S2. One paper used the term micro-team and was published in the UK.^26^ The authors offered no specific definition. Practices involved were free to define their own team model that could include any variety and number of professionals.

The most common term used was ‘teamlet’ (*n* = 16).^30^^–^^45^ When initially proposed in 2007, it described a ‘dyad relationship’ between a clinician and a health coach (health professionals whose expertise involves behaviour change and improving health outcomes by designing personalised goals and care plans for patients).^31^ Patients would be attended by both roles. The health coach complemented the clinician and expanded the consultation to provide more comprehensive care. The health coach would assist the patient in acquiring knowledge, skills, and confidence to self-manage health issues. Their role was emphasised when used to promote the self-management of chronic conditions.^43^

Publications from 2014 to 2019 described teamlets as adopting a larger team of four individuals comprising: a primary care practitioner (doctor, nurse practitioner, or physician associate), a registered nurse, a licensed practical nurse, and a clerical assistant (term used in US for receptionist) to provide comprehensive care.^34^^–^^41^^,^^44^^,^^45^

Huddles were described in seven papers.^30^^,^^31^^,^^36^^,^^41^^,^^43^^–^^45^ Although huddles do not have a standard definition, they are intended to be structured, brief (15 min), routine (multiple times a day), and face-to-face communication of a team’s full membership.^36^^,^^45^

The most common setting for papers was the Veterans Health Administration (VHA) (*n* = 9).^34^^,^^36^^–^^41^^,^^44^^,^^45^ The VHA offers care for US military veterans and certain family members.

### Question 2. Implementation

#### Deployment of resources

Staffing was reported as a key element in 11 studies.30,32,34,36–39,41,44,46,47 Flexibility in the team structure was described as an effective way to adapt to local resource constraints.^41^^,^^44^^–^^46^^,^^48^^,^^49^ The need for flexibility was balanced with the importance of role clarity.^37^^,^^38^^,^^41^^,^^44^^,^^45^^,^^47^ This meant clearly defined expectations in roles and responsibilities of all team members.^37^^,^^41^ Staff required training,^31^^,^^33^^,^^34^^,^^36^^–^^38^^,^^41^^,^^44^^,^^46^^,^^49^^,^^50^ which was conducted prior to and during implementation. Training involved education in how to operate as a micro-team and communication methods such as huddles.

Too much theory and terminology throughout training were viewed as unnecessarily rigid and conflated clinician responsibilities with administrative ones.^46^

Challenges to adequate staffing because of absences,^30^^,^^37^^,^^41^ high demand,^30^^,^^33^^,^^43^^,^^44^^,^^46^ or unmet need for staff expansion^36^ required cross-coverage from other teams.^37^^,^^44^^,^^47^

#### Culture of change

A cultural change of practice was described in 11 papers^30^^,^^41^^–^^47^^,^^49^^–^^51^ and included changes in values, perspectives, and working processes. The identification of practice members who would act as a ‘champion of change’ was mentioned in four studies.^30^^,^^46^^,^^49^^,^^50^ These individuals would celebrate positive achievements and use practice data to demonstrate improved health outcomes for patients to motivate participating GPs and sustain the implementation of micro-teams in the long term. For implementation to be a success, three papers described the importance of ‘buy-in’ from stakeholders of the intervention (that is, patients and those who worked in primary care).^42^^,^^46^^,^^47^ A paradigm shift towards a more patient-centred approach to care from a previously conventional doctor-centred approach was described in seven papers.^41^^–^^45^^,^^47^^,^^51^ Agency and locus of control were important factors to the practice staff experiencing this structural change.^36^^,^^37^^,^^46^^,^^50^^,^^52^ Internal agency provided visibility to valuable insights, perspectives, and contributions when team members felt in control of the practice change.^50^ If practices regarded the changes as an externally imposed demand on their time, they were more likely to withdraw or disengage from pilot studies.^46^ In contrast, external coaches advising how to successfully implement micro-teams were described as able to challenge entrenched hierarchies, mediate disagreements, and build consensus.^46^^,^^49^

#### Communication

Communication between team members was discussed in nine studies.^26^^,^^30^^,^^31^^,^^34^^,^^37^^,^^40^^,^^44^^–^^46^ Studies indicated the necessity for frequent and effective communication (for example, regular face-to-face meetings and huddles, often facilitated through technology) from leadership and transparency regarding prospective practice changes that related to the culture of change theme.^37^^,^^44^ Continuity and stability of team members benefited team communication.^32^^,^^33^^,^^44^^,^^47^ In turn, the cohesion of the team was reported to rely on regular communication.^36^^,^^45^

#### Development of understanding

Eleven studies highlighted the need for educational training to facilitate the adoption of micro-teams.^31^^,^^33^^,^^34^^,^^36^^–^^38^^,^^41^^,^^44^^,^^46^^,^^49^^,^^50^ Training would encompass how to operate effectively as a micro-team. In particular, training included awareness of individual roles and responsibilities of members within the micro-team. Mixed responses to training were reported, with certain individuals finding it ‘extremely valuable’ whereas others did not believe that concrete skills were imparted.^44^ It was suggested training should be conducted with team members to increase interoperability and provide a shared understanding. Orientation training was reported as a desirable introduction to micro-teams in defining roles and processes.^34^^,^^45^

The challenge of training part-time members of staff was highlighted.^47^ If a part-time individual was trained with one team cohort, the point was raised if and how much of the training might be repeated in this circumstance. Inadequate training was perceived as a barrier in five studies.^36^^,^^38^^,^^40^^,^^41^^,^^44^

### Question 3. Care organisation

#### Aligned ethos of team

Establishing a mutual set of expectations among the organisational and clinical leaders was described as a beneficial outcome in four papers.^32^^,^^46^^,^^50^^,^^51^

Leaders who communicated their vision of transformation, set expectations, and committed resources were described as a critical component of practice redesign.^50^ In teams with less collaboration, certain members were described as being difficult to work with or unenthusiastic towards their work.^32^

#### Sustainable team interrelationship

Team cohesion was described in nine studies.^32^^,^^34^^,^^37^^,^^38^^,^^41^^,^^43^^,^^44^^,^^46^^,^^47^ Establishing and maintaining team continuity was reported to contribute to sustaining relationships between healthcare team members and consequently improved ongoing relationships with patients.^33^^,^^44^^,^^46^^,^^47^

#### Patient panel integrated into the team

Teams were assigned a specific panel of patients in nine papers.^31^^,^^32^^,^^37^^,^^41^^,^^43^^–^^45^^,^^47^^,^^50^ These papers reported that patient panels did not cover a specific disease or condition, but followed a generalist care model. Continuity was maintained by ensuring team members always cared for a patient on their team’s panel.^31^^,^^44^ In practice, staffing absences made this challenging to achieve.^31^

One paper described the involvement of patients as stakeholders in the redesign process of the practice.^50^ Patients viewed this engagement positively, helping to inform and shape their care.

A common benefit of the teamlet model was providing greater opportunities for patient education through the health coach role.^30^^–^^33^^,^^38^^,^^40^^,^^42^^–^^44^^,^^47^ The health coach assisted the patient in gaining knowledge, skills, and the ability to self-manage health issues.

One paper acknowledged the benefit of having a separate team that would focus on walk-ins to reduce the burden of unanticipated appointments.^44^ Practices with fewer walk-ins and more planned visits found it easier to develop the roles and responsibilities of team members.^41^^,^^44^

In three papers, patients were allocated to teams who shared their language and cultural background.^33^^,^^42^^,^^43^ By sharing a common culture, staff could gain valuable insight into patients’ daily lives.^33^^,^^43^

One paper raised concerns regarding potential problems with continuity delivered by a team from the patient’s perspective.^48^ A patient loyal to a particular healthcare professional may delay seeking help until that team member is available to their own detriment.^48^ In addition, familiarity may breed complacency and a serious diagnosis may be missed. Furthermore, continuity may not necessarily guarantee an effective relationship between the patient and healthcare provider.^48^

### Quality assessment

The quality assessment did not determine whether a paper was included or not, but was used to determine the relevance and trustworthiness of data for analysis. A summary of quality assessment using the Mixed Methods Appraisal Tool (MMAT) is shown in Supplementary Table S3.^53^

## DISCUSSION

### Summary

Primary care organisations can have an impact on the nature and quality of patient care. This review contributes to current debates surrounding the organisation of care and how this can have an impact on the experiences and outcomes of patients and staff in both the UK and international settings. The analysis identifies the promising potential of micro-team implementation through key knowns. Key unknowns surround patients’ perspectives and financial considerations. The evidence from this review contributes to current debates surrounding care organisation and how this can have an impact on the experiences and outcomes of patients and staff. For an overview of what has been established from this review and what remains unclear, see Box 1.

**Box 1.** What is known about the topic and what remains unclear

**What is known**	**What remains unclear**
Effective team communication matters, huddles are an example of this in practice Sustainable team culture matters — development of interoperability and cohesion, achieved through stable teams Clarity of individual roles and responsibility within the team through education is essential Roles should be flexible and staff willing to take on new responsibilities Affiliation to the wider practice team should be retained or a feeling of responsibility for all patients may be lost	Does continuity offered between a patient and individual or patient and team differ? Does it matter which individual in the micro-team offers continuity? The applicability of international findings to the UK practice setting Patient experiences and outcomes Financial and economic implications for the sustainability of the model The impact on patient access to a preferred clinician and appointments more generally Distinctions between models of care for acute and chronic problems, and the interface between the two Would a patient prefer to consult separate individuals for these?

The concept of micro-teams is described under a variety of terms and team compositions. Micro-teams are embedded within the wider practice team, working in conjunction and sharing specialist roles between team groups. Micro-teams may involve an increased number of staff for each consultation. This implies potential fiscal consequences, which no study has examined to date. It is anticipated that the micro-team approach would decrease the frequency of consultations a patient requires, thus a potentially positive step towards sustainable healthcare goals.^54^

The optimum context for the implementation of micro-teams is controversial. Most studies report their introduction within a generalist model of care. Accommodating unscheduled appointments is challenging for the micro-team model. Micro-teams were easier to introduce in practices with full-time staff working fixed timetables. However, the features that made implementation easier in these examples, such as continuity that established familiarity and team stability, could be embedded into teams with part-time members.

Although 21 papers were empirical, few provided rich, detailed descriptions of the patient perspectives. There was a minimal acknowledgement of the rationale to focus on implementation, rather than patient and healthcare professional outcomes.

### Strengths and limitations

The method process of this review is clearly laid out. The underlying principles of systematicity and methodological rigour are maintained by ensuring transparency and replicability. Patient representation and stakeholder collaboration have been key strengths. This input helped ensure the relevancy of the findings and proposed recommendations.

Based on the quality assessment, several included studies had a limited analysis of methodology and were susceptible to bias. It was decided to retain these studies as the aim of this review was to analyse all relevant available literature and not to determine an effect size. Given the range of descriptions of micro-teams, it is possible that included search terms neglected relevant citations; however, no further appropriate terms were found during the analysis of papers.

### Comparison with existing literature

The findings of this review regarding micro-teams are consistent with the drive towards patient-centred care (PCC) and the personalised care initiatives outlined in the NHS Long Term Plan and the Royal College of General Practitioners innovation programme.^55^^,^^56^

Micro-teams have the potential to offer PCC through improved continuity, with patients seeing a member of a particular team and maintaining accessibility if members of the team are available at different times. PCC has been positively associated with the physical and social wellbeing of patients in the primary care setting.^57^^–^^60^ The NHS has incorporated PCC into its comprehensive model of personalised care to establish *‘intensive and integrated approaches to empowering people with more complex needs to have greater choice … over the care they receive’*.^61^

Micro-teams offer the potential for continuity between the patient and a team of healthcare professionals. There is a key distinction, however, between the continuity with an individual clinician and the continuity provided by a team. Continuity reduces morbidity and mortality. It was defined by Pereira Gray *et al* as *‘repeated contact between an individual patient and a doctor’*.^16^ A further systematic review by Baker *et al* defined continuity of care as *‘the care of individuals … over time’*.^17^ This definition has applicability to micro-teams, although effective and sustained communication is necessary to facilitate continuity, potentially through huddles.

Separate micro-teams caring for a particular panel of patients were described in this review as embedded in a wider practice team. There is a hypothesised danger of a ‘silo-mentality’ that has been defined as keeping information or methods of practice hidden from others in the broader team.^62^ The responsibility of patients outside a team’s panel may be questioned and competition between teams may arise. For example, if a patient requires a consultation for an acute health concern, but there is limited availability to be seen by their customary micro-team, there is a question whether they could be seen more immediately by a different micro-team at the practice. Each team must have the flexibility to adapt to the needs of various patient cohorts, maintaining a broader vision of organisational culture.

### Implications for research and practice

As general practice expands in the UK it is an intriguing space to explore how care delivery is organised. The NHS Long Term Plan describes the move to integrated care systems (ICSs) and primary care networks (PCNs).^55^ The significant challenges of practice expansion and cross-working that PCNs and ICSs have presented are coupled with the recent adjustments to care caused by COVID-19, such as the increased volume of remote consultations.^63^^–^^66^ Given the focus on increasing practice size to improve quality of care and generate efficiencies, practice organisation is an important area to consider.

The contribution of UK publications to this review is modest, with only two papers.^26^^,^^46^ Internationally, this review has highlighted the need for further information and studies about the impact of micro-teams on costs, granular patient experience, access, and continuity. Further research is needed to inform the applicability and transferability of these international results to the UK primary care setting.

## Data

The dataset generated and analysed during the study is not publicly available but is available from the corresponding author on reasonable request.
